# Rates of Cancer, Non-curative Resection, Adverse Event and Surgery After Colonic Endoscopic Submucosal Dissection (ESD)—Results from a Large International Multicenter Study

**DOI:** 10.1007/s10620-025-09621-8

**Published:** 2025-12-16

**Authors:** Rahul Karna, Jonathan Colón Sánchez, Kevan Josloff, Tammy Tran, Kasenee Tiankanon, Saowanee Ngamruengphong, Elisabet Maristany Bosch, Georgios Kalopitas, Edward John Despott, Alberto Murino, Shaimaa Elkholy, Mohamed El Sherbiny, Karim Essam, Hany Haggag, Abeer Awad Abdallatef, Kerolis Yousef, Rossella Maresca, Federico Barbaro, Galen Leung, Frances Dang, Amirali Tavangar, Jason Samarasena, Ahmed Saeed, Sherif Andrawes, Yutaka Tomizawa, Mohammad Bilal, Kartik Sampath, Yasi Xiao, Faisal Kamal, Thomas Kowalski, Alexander Schlachterman, Anand R. Kumar

**Affiliations:** 1 Division of Gastroenterology & Hepatology, University of Minnesota, Minneapolis, MN 55414 USA; 2 Department of Internal Medicine, Thomas Jefferson University Hospital, Philadelphia, PA USA; 3 Division of Gastroenterology & Hepatology, Department of Medicine, Thomas Jefferson University Hospital, Philadelphia, PA USA; 4 Division of Gastroenterology & Hepatology, Johns Hopkins Medicine, Baltimore, MD USA; 5 Division of Gastroenterology, Department of Medicine, Faculty of Medicine, Chulalongkorn University and King Chulalongkorn Memorial Hospital, Bangkok, Thailand; 6 Institute for Liver & Digestive Health, UCL (University College London), The Royal Free Hospital, London, UK; 7 Gastroenterology Division, Internal Medicine Department, Faculty of Medicine, Cairo University Kasr Alainy, Cairo, Egypt; 8 Digestive Endoscopy Unit, Fondazione Policlinico Universitario Agostino Gemelli IRCCS and Università Cattolica del Sacro Cuore, Rome, Italy; 9 Division of Gastroenterology & Hepatology, New York University Langone Health, New York City, NY USA; 10 Division of Gastroenterology, University of California Irvine, Orange, CA USA; 11 Division of Gastroenterology, Research Medical Center, Kansas City, MO USA; 12 Division of Gastroenterology and Hepatology, Staten Island University Hospital-Northwell Health, Staten Island, NY USA; 13 Division of Gastroenterology, University of Washington, Seattle, WA USA; 14 Division of Gastroenterology & Hepatology, University of Colorado Anschutz Medical Campus, Aurora, CO USA; 15 Division of Gastroenterology, New York Presbyterial Hospital, Weill Cornell Medical Center, New York City, NY USA

**Keywords:** Colon cancer, Endoscopic submucosal dissection, Endoscopic resection

## Abstract

**Background and Aims:**

Endoscopic submucosal dissection (ESD) is preferred over endoscopic mucosal resection (EMR) for resection of colon cancer with suspected superficial invasion. Data on appropriate utilization of ESD such as rates of cancer and non-curative resection (NCR) are under-reported.

**Methods:**

Retrospective multicenter study of 547 consecutive colonic lesions undergoing ESD was performed. Outcomes were rates of cancer, NCR, surgery and predictors of NCR.

**Results:**

Of all lesions, histopathology demonstrated cancer in 12% (n = 66). Overall, NCR was seen in 59.1% (n = 39) patients and 24.6% (n = 135) lesions contained high-grade dysplasia. For NCR, patients underwent surgery in 7.6% (n = 3) and adverse events were observed in 8.8% (n = 48).

**Conclusions:**

In our large multicenter Western cohort, the pathology was found to be benign in most of the colon ESDs and there was high NCR for resected lesions with cancer. The overall surgery rate, however, remained low. This study highlights the need to refine lesion selection criteria while continuing to optimize ESD technique to match the efficiency and safety of EMR.

**Supplementary Information:**

The online version contains supplementary material available at 10.1007/s10620-025-09621-8.

## Introduction

Colon cancer develops through well defined “adenoma-carcinoma sequence” and colonoscopy can potentially detect adenomatous polyps with cancer invading submucosa. Management of T1 colon cancer has evolved in recent years with advancement in endoscopic resection (ER) techniques. T1 colon cancer with low risk of lymph node metastases (LNMs) can undergo ER, while those with higher risk should undergo surgical resection to allow simultaneous lymph node sampling [1].

The optimal management strategy of colon cancer with suspected superficial submucosal invasion requires consideration of risk of residual cancer and LNMs with ER weighing against the risk of adverse events (AEs) with surgery. En-bloc resection (generally achieved with endoscopic submucosal dissection, ESD) is the preferred resection strategy for colon cancer with features of superficial submucosal invasion due to low risk of LNMs. However, most large non- pedunculated colon polyps (LNPCPs) without submucosal invasion can be safely resected by endoscopic mucosal resection (EMR) [2]. ESD provides en-bloc specimen for accurate depth of invasion assessment but requires technical expertise and is limited to centers of expertise. A randomized trial, demonstrated higher en-bloc resection rate (93.8% vs 6.6%), decreased six month recurrence (0.6% vs 5.1%) but higher AEs (35.6% vs 24.5%) including perforations (5.7% vs 2.2%) with ESD compared to EMR for LNPCPs [3]. Currently, the technique for ER of LNPCPs is based upon available expertise, institutional protocols and pre-operative optical diagnosis of lesions.

Here, we aim to assess histopathological, surgical outcomes and AE profile of colonic ESD, specifically focusing on patients with cancer, in the largest multicenter Western cohort, to our knowledge.

## Materials and Methods

This is an international multicenter (11 centers; 8 US, 1 UK, 1 Italy and 1 Egypt) retrospective study of patients who underwent colonic ESD between 2017 and 2024. Institutional review board approval was obtained at primary site, and participating centers.

All consecutive adult patients who underwent colonic ESD as primary or secondary modality of a previously manipulated lesions were included. Patients who underwent ESD for rectal lesions were excluded from our study, due to differences in the risk of deep submucosal invasion, delayed adverse events, technical complexity and differences in learning curve for practicing endoscopist compared to colonic ESD. Demographic details, history of prior interventions, lesion morphology and characteristics, ESD techniques, procedure details, final histopathology and follow up data were collected. Primary outcome was rates of colon cancer and non-curative resection (NCR). NCR was defined as colon cancer with one of the following: deep submucosal (> 1 mm) invasion (DSI), positive or indeterminate margins, poor differentiation, lympho-vascular invasion, and high tumor bud score. Those not meeting any of the criteria were considered as curative resection. eNCR (extended NCR) was defined as colon cancer with one of the following: positive or indeterminate margins, poor differentiation, lympho-vascular invasion, and high tumor bud score, after excluding DSI as one of the criteria. Secondary outcome measures included rate of adverse events (AEs), surgical resection rate for NCR or AEs and periprocedural predictors of NCR.

Descriptive statistics and comparative analysis were performed using Chi-square (or Fisher exact test) for categorical variables. Statistical significance was set at a p-value of < 0.05. SPSS v30.0 (IBM SPSS Statistics for Windows, IBM Corp., Armonk, NY) was used to perform analyzes.

## Results

### Study Characteristics

A total of 547 patients with mean age 64.2 ± 11.6 years were included in the study. 10.6% patients were on anticoagulants or antiplatelet agents (excluding aspirin). Prior intervention was noted in 14.1% (n = 77) with either snare resection, EMR or surgical anastomosis at polyp site. Lesions were more commonly located in proximal colon (n = 333, 60.8%). Majority of the lesions were classified as LST-granular mixed (n = 144, 37.6%), NBI international colorectal endoscopic classification (NICE) 2 (n = 351, 94.1%) and Japanese NBI Expert Team (JNET) 2A (n = 221, 55.3%) or JNET 2B (n = 161, 40.25%). Submucosal fibrosis was present in 50.6% (277/547). Supplementary Table 1 demonstrates description of polyp location, morphology and ESD technique used.

### Study Outcomes

En-bloc resection was achieved in 86.1% (471/547). Mean procedure time was 159.3 min. Nearly half (46.4%, n = 254) of the patients were hospitalized post-ESD for mean 1.95 days. One-fourth of the patients (n = 135) had high-grade dysplasia and 12.1% (n = 66) had cancer (Table 1). Of the lesions demonstrating cancer, pathology demonstrated NCR in 59.1% (n = 39) patients. Patients were deemed to have NCR due to DSI (> 1 mm) in 53% (n = 35), positive/indeterminate margins in 31.8% (n = 21), lympho-vascular invasion in 24.2% (n = 16) and high tumor budding score in 6% (n = 4). Using the extended curative criteria (i.e. excluding DSI as a high risk feature), the eNCR did not significantly decrease and was seen in 50.8% (n = 33) of the patients with cancer. Polyp location, extent, morphology, presence of submucosal fibrosis, prior intervention, use of traction or hybrid techniques were not significantly associated with NCR. (Table 2) For patients with NCR, 3 (7.6%) underwent surgery and the remaining were followed conservatively. Patients were followed for a median of 12 (3–46) months after ESD.

**Table 1:** Rates of cancer, non-curative resections, and surgery in analytic cohort

Variable	n (%)
High-grade dysplasia	137 (25%)
Colon cancer	66 (12.1%)
- Deep (> 1 mm) submucosal invasion	35 (53%)
- Positive/Indeterminate margins	21 (31.8%)
- Lympho-vascular invasion	16 (24.2%)
- High tumor bud score	4 (6%)
Right colon cancer (n = 260)	27 (10.4%)
Proximal colon cancer (prox. to splenic flexure, n = 330)	35 (10.6%)
Distal colon cancer (distal to splenic flexure, n = 217)	31 (14.3%)
Non-curative resections for colon cancer	
- All cancer (n = 66)	39 (59.1%)
- Right colon cancer (n = 27)	13 (48.1%)
- Proximal colon cancer (n = 35)	18 (51.4%)
- Distal colon cancer (n = 31)	21 (67.7%)
Surgery	
- For colon cancer (n = 66)	3 (4.5%)
- For adverse events (n = 48)	7 (14.5%)

**Table 2:** Predictors for non-curative resection among lesions with histopathology demonstrating colon cancer

Variable	Non-curative resection (n = 39)	Curative resection (n = 27)	p-value
Previous EMR or surgery	4 (10.3%)	4 (14.8%)	0.71
Proximal colon lesions	18 (46.2%)	15 (55.6%)	0.45
Right colon lesions	13 (33.3%)	13 (48.1%)	0.23
Paris 0-Is or Isp lesions	27 (69.2%)	14 (51.9%)	0.15
Traction used	10 (25.6%)	5 (18.5%)	0.50
Submucosal fibrosis	25 (64.1%)	13 (48.1%)	0.20
Hybrid ESD en-bloc resection	3 (9.4%)	1 (4.2%)	0.63
NICE 3	8 (30.8%)	7 (31.8%)	0.94
JNET 3	8 (20.5%)	4 (14.8%)	0.53
Age (mean ± SD)	68.0 ± 10.2	65.7 ± 10.2	0.37
Circumferential involvement (mean)	35.0 ± 14.5	38.9 ± 20.8	0.44
Lesion size (mean ± SD)	43.5 ± 21.7	46.3 ± 26.5	0.64
Procedure time (mean ± SD)	131.5 ± 75	144.4 ± 103	0.50

*EMR* endoscopic mucosal resection, *ESD* endoscopic submucosal dissection, *NICE* NBI international colorectal endoscopic classification, *JNET* Japanese NBI Expert Team

### Adverse Events

Adverse events (AEs) were seen in 48 patients (8.8%) in our study. Perforations in 15 patients (2.7%) and bleeding in 14 patients (2.6%) were the most common reported AEs. Perforations were managed surgically in 46.7% (n = 7), and the rest were managed endoscopically (n = 4; 26.7%) and conservatively (n = 4; 26.7%). Severity of AEs were mild in 58.1% (n = 25), moderate in 23.2% (n = 10) and severe in 18.6% (n = 8) as per American Society of Gastrointestinal Endoscopy (ASGE) Lexicon classification [4]. In patients with cancers, 7.6% (n = 5) had a delayed AE (DAE) within 2 weeks of ESD, consisting of perforation (n = 3), abdominal pain (n = 1) or respiratory failure (n = 1). Patients with perforation (n = 3) were managed with surgery, endoscopically and conservatively one each, respectively. Table 3 summarizes AEs in our study cohort.

**Table 3:** Summary of adverse events after ESD in our cohort

Variable	n (%)
ESD adverse events in all lesions (n = 547)	48 (8.8%)
- Intraprocedural perforation	4 (0.7%)
- All delayed adverse events within 2 weeks	44 (8%)
- Delayed bleeding within 2 weeks	14 (2.6%)
- Delayed perforation within 2 weeks	15 (2.7%)
ESD adverse events in colon cancer (n = 66)	5 (7.6%)
- Intraprocedural perforation	0 (0%)
- All delayed adverse events within 2 weeks	5 (7.6%)
- Delayed bleeding within 2 weeks	0 (0%)
- Delayed perforation within 2 weeks	3 (4.5%)

## Discussion

Our large international multicenter study demonstrates that majority (87.9%) of colonic ESD are performed for benign lesions. Overall 12.1% of resected lesions were colon cancer, with majority (59.1%) not achieving curative resection. Even with extended curative criteria, the NCR remained high at 50.8%. The overall AE rate was 8.8% and rate of surgery for cancer due to NCR and AE was 7.6%, both relatively low in our study.

ESD is becoming popular in the Western countries for resection of LNPCPs. Japanese guidelines recommend ESD for fibrotic lesions, residual early carcinoma after previous manipulation and lesions ≥ 20 mm with submucosal invasion [5]. In our study, the majority of the lesions (84%) did not have a prior history of manipulation. Assessment of depth of invasion of lesion is based on the evaluation of polyp morphology, surface vascular pattern and pit patterns. However, the optical detection of submucosal invasion is challenging with significant inter-observer variability [6]. In our study, 12% of lesions had evidence of submucosal invasion after ER, with majority not achieving curative resection emphasizing US Multi Society Task Force recommendations on the need for improvement in depth of invasion assessment criterion and standardization in training [1]. In comparison, previous studies from Japan, have established curative resection rates upto 90%, along with lower adverse event rate of 3.8% [7]. Although direct comparisons from Japanese studies can’t be drawn as ESD is currently still in evolving phase in the West, and it is a well-established technique for early cancer removal in Japan, several lessons can be implemented. The current criteria involving NICE, JNET and KUDO classification system, have not been fully adopted due to variable training and availability of high-level magnification colonoscopes. European Society of Gastrointestinal Endoscopy recently recognized these lacunae and published a curriculum on optical diagnosis training, however, widespread adoption across training programs is still lacking [8]. There are several barriers with the use and reliance of optical diagnosis during ESD. In addition to inter-observer variability, detection of a small cancer within a large polyp can be challenging and sometimes not possible. Moreover, as we note in our study, the optical diagnosis pre-ESD or the morphology of the polyp did not predict who achieved NCR. Japanese ESD training pathway is well standardized following the master-apprenticeship model, and high prevalence of gastric cancer allows trainees to learn less complex gastric ESD prior to performing colonic ESD [9]. The pathway allows intensive cognitive and procedural training, and mastery of ESD techniques in Japanese endoscopists. Availability of training pathways in the West for endoscopists interested in learning ESD techniques would be paramount. Due to low prevalence of gastric cancer, Japanese model can’t be strictly implemented in the West [9]. Endoscopists in the West should train on rectal and esophageal lesions prior to performing colon ESD. However, with continuous improvement in techniques, and increasing availability of training opportunities in the West, ESD outcomes are expected to improve.

Despite advancement in ESD training, piecemeal EMR remains an acceptable approach for removal of large benign polyps. Polyps with suspected superficial invasion, underlying fibrosis or previously manipulated lesions should undergo ESD. An incremental cost effectiveness analysis performed for large lateral spreading colorectal lesion reported selective ESD to be the most cost effective for vast majority of lesions, rather than universal ESD (performing ESD for all LNPCPs to obtain en-bloc specimen to achieve precise histopathological analysis and reduce recurrence) or EMR [10]. Universal ESD for lesions that could be suitable for EMR is not a cost-effective or time-saving strategy [10]. Time taken to complete an ESD in our study ranged from 50 to 908 min. The resection speed currently in the US lags behind the Asian countries. Among Western countries, France has pioneered the universal ESD strategy for all LNPCPs with resection speeds over 15 mm^2^/min. with use of saline immersion technique combined with traction and have also demonstrated reproducibility of these speeds and safety in trainees [11]. The same group with novel traction devices have reported dissection speeds around 60 mm^2^/min [12]. At these dissection speeds and low recurrence rates[3], ESD procedure can potentially be transformed from a cancer-curative to a reduced-recurrence procedure that could also reduce colectomy rates and lengthen surveillance intervals. Furthermore, en-bloc specimen obtained after ESD has diagnostic utility, particularly in NCR where histological staging can help guide surgical resection. With recent approval of a CPT code for ESD in the US, the technique’s popularity will likely further increase. The strategy of universal ESD vs selective ESD will have to consider several factors, including disease burden, resources and expertise availability, cost and reimbursement in the West, however, improvement in optical diagnosis would be imperative prior to deciding strategy at a national level.

Our study is limited by the possibility of lesion selection bias, lack of data on endoscopists experience, center volume, cost-analysis, lack of comparison group (EMR), and long term follow up data. There was likely significant heterogeneity between centers in terms of indication of ESD, endoscopists training and experience, available devices and tools, and center volume. Our data does not demonstrate polyp location, size, morphology or presence of fibrosis as a predictor of NCR, however, due to small number of patients (n = 39), this may reflect a lack of power, rather than true effect. Even though, over 50% of the polyps with cancer did not meet curative criteria, the overall surgery rate remained low. This could be due to patient choices or comorbidities or performance status which was unfortunately not available in our dataset. While this may have affected the rate of surgery, the overall NCR is still over 50% for polyps with cancer who underwent ESD. ESD technique, devices and strategy may also have evolved over the study period, leading to improved efficiency and safety. The study did not capture details of techniques, devices and endoscopists experience over the study period. However, our study capturing real-world data from 11 expert ESD centers, highlights current practice patterns and provides significant insights into practice patterns with suggested areas of improvement.

In conclusion, polyp selection for colonic ESD remains controversial. Pathology on majority of the colon ESDs was found to be benign in our study. Among 12% cancers, over half did not achieve curative resection even with extended criteria. These observations raise questions on the selection of colon polyps for ESD and if ESD can reliably achieve curative resection for colon polyps with cancer. There is a need to further optimize lesion selection for colonic ESD in the West. Compared to Asia, differences in expertise and training systems likely contribute to discrepancies in outcomes compared to the East, particularly in terms of resection speed and non-curative resection rates. The study findings underline a need for national and regional registries to monitor outcomes, promote standardized training, and reduce variability among distinct geographic clusters.

## Conflict of Interest

FB: Consultant for Olympus; YT: Consultant for Boston Scientific, Medtronic; MB: Consultant for Boston Scientific, Steris Endoscopy and paid speaker for Cook Endoscopy; KS: Consultant for ConMed, Lumendi; TK: Consultant for Boston Scientific; AS: Consultant for Boston Scientific, Olympus, FujiFilm, Lumendi, Laborie; ARK: Consultant for Boston Scientific, Olympus, ConMed, Pentax.

## Supplementary Information

Below is the link to the electronic supplementary material.

Supplementary file1 (DOCX 18 KB)

## Data Availability

No datasets were generated or analyzed during the current study.

## Abbreviations

ESD: Endoscopic submucosal dissection
NCR: Non-curative resection
CRC: Colorectal cancer
LNM: Lymph node metastases
LNPCP: Large non- pedunculated colon polyps
EMR: Endoscopic mucosal resection
DSI: Deep submucosal invasion
AE: Adverse events
LST: Lateral Spreading Tumor
NBI: Narrow band imaging
NICE: NBI international colorectal endoscopic classification
JNET: Japanese NBI Expert Team
