# Supplementary material for: Rates of Cancer, Non-curative Resection, Adverse Event and Surgery After Colonic Endoscopic Submucosal Dissection (ESD)—Results from a Large International Multicenter Study
Source: Dig Dis Sci. 2025 Dec 16;71(6):2508–13. doi: 10.1007/s10620-025-09621-8 (PMC13357622; doi:10.1007/s10620-025-09621-8)
Supplement: Supplementary file 1 — Supplementary file1 (DOCX 18 KB) [file 10620_2025_9621_MOESM1_ESM.docx]

Supplementary Table 1: Baseline characteristics of patients undergoing endoscopic submucosal dissection

| Variable | N (%) |
| --- | --- |
| Primary Indication  Polyp  Residual polyp after prior snare, EMR or surgery  Others | 460 (84.1%)  77 (14.1%)  10 (1.8%) |
| Lesion Location  Cecum  IC valve  Ascending colon  Hepatic flexure  Transverse colon  Splenic flexure  Descending colon  Sigmoid colon | 62 (11.3%)  20 (3.6%)  137 (25.1%)  35 (6.4%)  71 (12.9%)  12 (2.2%)  50 (9.1%)  160 (29.2%) |
| Polyp size, mean+/-S.D., in mm | 43.15+/-20.23 |
| Degree of circumferential involvement  <50%  ≥ 50 to <75%  ≥75 to <90%  ≥90% to <100%  100% | 301(75.6%)  78 (19.6%)  8 (2.01%)  7(1.8%)  4 (1.0%) |
| Paris Classification  Is/Isp  IIa  IIb | 287 (63.5%)  92 (20.4%)  73 (16.15%) |
| Subtypes of LST classification  LST-G homogenous  LST-G mixed  LST-NG  LST-NG pseuododepressed | 112 (29.2%)  144 (37.6%)  89 (23.2%)  38 (0.99 %) |
| Narrow Band Imaging Polyp Classification  NICE 1/2/3  JNET 1/2A/2B/3 | 4 (1.1%)/351(94.1%)/18(4.8%)  4 (1%)/221(55.3%)/161(40.25%)/14(3.5%) |
| Submucosal Fibrosis  F0  F1  F2 | 269 (49.2%)  140 (25.6%)  138 (25.2%) |
| Details of ESD technique used  Traditional  Tunnel/Pocket creation  Saline immersion  Hybrid EMR-ESD  combination  Others | 238 (43.9 %)  98 (18.1%)  16 (2.9%)  78 (14.4%)  109 (20.1%)  4 (0.7%) |
| Traction utilized  Clip band/Clip line or floss/Others | 151 (27.6%)  72/9/49 |
| ESD knife  Dual J  Hybrid  Proknife  Multiple  Others | 215 (39.5%)  91(16.7%)  38 (6.9%)  48(8.8%)  152 (27.9%) |
| Prophylactic hemostasis | 308 (56.4%) |
| Prophylactic closure  Though the scope hemoclips/X-tack/Overstitch/Mantis/DAT/Combination | 350 (63.9%)  203/43/69/15/1/64 |
| Application of topical hemostatic agent  Purastat/Endoclot/Nexpowder/Others | 69 (12.6%)  60/5/2/2 |

Abbreviations: LST: lateral spreading tumor; EMR: endoscopic mucosal resection; NICE: Narrow Band Imaging International Colorectal Endoscopic Classification; JNET: Japan Narrow Band Imaging Expert Team; ESD: endoscopic submucosal dissection
